# A Deep Learning Segmentation Approach in Free-Breathing Real-Time Cardiac Magnetic Resonance Imaging

**DOI:** 10.1155/2019/5636423

**Published:** 2019-07-30

**Authors:** Fan Yang, Yan Zhang, Pinggui Lei, Lihui Wang, Yuehong Miao, Hong Xie, Zhu Zeng

**Affiliations:** ^1^Key Laboratory of Biology and Medical Engineering, Guizhou Medical University, Guiyang 550025, China; ^2^School of Biology & Engineering, Guizhou Medical University, Guiyang 550025, China; ^3^Department of Radiology, The Affiliated Hospital of Guizhou Medical University, Guiyang 550004, China; ^4^Key Laboratory of Intelligent Medical Image Analysis and Precise Diagnosis of Guizhou Province, School of Computer Science and Technology, Guizhou University, Guiyang 550025, China

## Abstract

**Objectives:**

The purpose of this study was to segment the left ventricle (LV) blood pool, LV myocardium, and right ventricle (RV) blood pool of end-diastole and end-systole frames in free-breathing cardiac magnetic resonance (CMR) imaging. Automatic and accurate segmentation of cardiac structures could reduce the postprocessing time of cardiac function analysis.

**Method:**

We proposed a novel deep learning network using a residual block for the segmentation of the heart and a random data augmentation strategy to reduce the training time and the problem of overfitting. Automated cardiac diagnosis challenge (ACDC) data were used for training, and the free-breathing CMR data were used for validation and testing.

**Results:**

The average Dice was 0.919 (LV), 0.806 (myocardium), and 0.818 (RV). The average IoU was 0.860 (LV), 0.699 (myocardium), and 0.761 (RV).

**Conclusions:**

The proposed method may aid in the segmentation of cardiac images and improves the postprocessing efficiency of cardiac function analysis.

## 1. Introduction

Free-breathing cardiac magnetic resonance (CMR) cine imaging techniques have been developed for the evaluation of cardiac function [1–5]. It is an accurate and reproducible technique for chamber volume, myocardial mass, and stroke volume measurements [5]. Compared with breath-hold CMR cine imaging, it has a short acquisition time and eliminates the unnecessary breath-hold stage. It is beneficial for children and patients who are unable to hold their breath during data acquisition. However, the postprocessing of free-breathing CMR cine imaging is time-consuming and laborious. Although some commercial software could automatically segment the left ventricle (LV) blood pool, LV myocardium, and right ventricle (RV) blood pool contours of end-diastole (ED) and end-systole (ES) frames, manual adjustment of the segmented contour is still required by an expert. However, the procedure could introduce intraobserver and interobserver variability [6]. Hence, a fully automatic segmentation method of ED and ES frames is necessary for improving the postprocessing efficiency of the free-breathing CMR cine imaging.

The segmentation process of cardiac imaging was previously divided into two stages, i.e., localization and segmentation. For the localization task, some studies used variance image [7], the Fourier Transform [8], and the circular Hough transform [9] to locate the heart. Due to the diaphragm motion in free breathing CMR cine imaging, these methods cannot be applied directly for the heart localization, especially for the apex slice. For the segmentation task, the level set was previously widely used for the LV, myocardium, and RV [10–13]. Other methods include threshold, pixel classification, cardiac atlas, shaped registration, and active shape model, among others [6, 14–19]. However, most algorithms require prior information and manual operation. An alternative automatic method to locate and segment the heart from the cardiac image is to use deep learning techniques.

**Figure 1:**
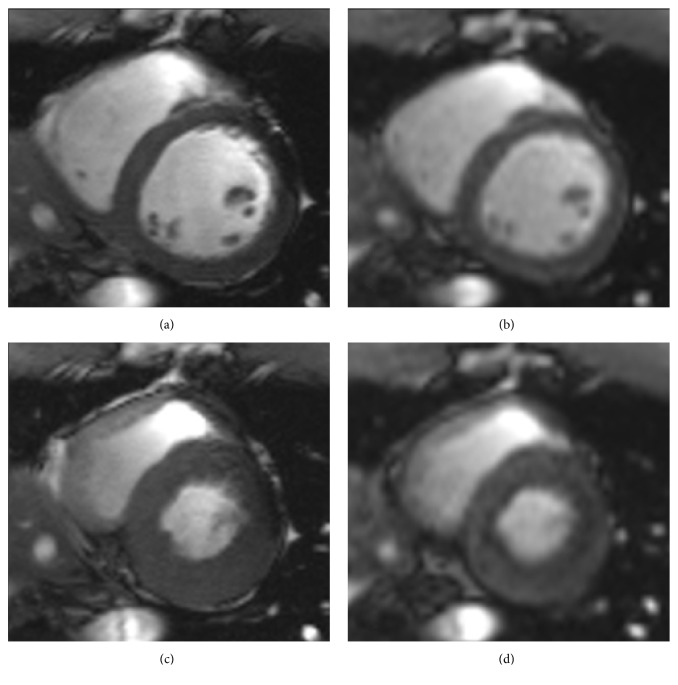
Comparison of breath-hold CMR data (a, c) and free-breath CMR data (b, d), where (a, b) are ED frames and (c, d) are ES frames.

Recently, deep learning approaches have been widely used in medical image segmentation, especially for CMR images [20–35]. Bernard et al. [20] summarized all the types of segmentation methods using deep learning. Avendi et al. [21] presented a combined deformable model and deep learning method for LV segmentation. Ngo et al. [22] proposed a deep learning method combined with the level set for LV segmentation. Tan et al. [23] designed a regression network on the segmentation of short-axis LV. Isensee et al. [24] used an ensemble of modified 2D and 3D U-Net to tackle the segmentation. Baumgartner et al. [25] tested various convolution neural networks with hyperparameters. Zotti et al. [26] proposed an extension of the U-Net for the cardiac segmentation and an automated analysis method for CMR images [27]. Vigneault et al. [28] presented a novel network for localization and semantic segmentation of cardiac images. Zheng et al. [29] applied a novel variant of U-Net for the cardiac segmentation on short axis MRI image. Khened et al. [30] utilized multiscale residual DenseNets for cardiac segmentation. Zhang et al. [31] proposed a novel deep network for the segmentation of myocardial infarction. Bai et al. [32] used a fully convolutional network for CMR image analysis. Qin et al. [33] proposed a motion estimation and segmentation method for cardiac images. Oktay et al. [34] developed neural networks to cardiac image enhancement and segmentation. Romaguera et al. [35] studied the myocardial segmentation with deep learning approach. These methods were successful for cardiac segmentation. However, the image quality of free-breathing CMR imaging is lower than the breath-hold CMR imaging (see Figure 1) [36]. The deep learning techniques for the accurate segmentation of free-breath CMR data remain a challenge.

As mentioned above, the development of a fully automatic method for the segmentation of free-breathing CMR images could assist experts in analyzing cardiac function. In this paper, we propose a one-stage deep learning network based U-Net [37] for the segmentation of the heart. The LV, LV myocardium, and RV were directly segmented by using presented deep learning model. The ACDC data were used for training, and free-breathing CMR data were used for validation and testing. We also used a random augmentation data strategy during training. The data were only augmented during the training process and were not stored, making it faster than data augmentation before training. Furthermore, we proposed an improved loss function to yield higher segmentation accuracy. The proposed method is validated and tested on free-breathing CMR data. The experimental results validate its accuracy for cardiac segmentation. The layout of the paper is as follows: The datasets are introduced in detail in Section 2; the methods are presented in Section 3; Section 4 demonstrates the experimental results; in Section 5, we discuss and analyze the results; and the conclusions are given in Section 6.

## 2. Materials

### 2.1. Free-Breathing CMR Data

The study was approved by the institutional Review Board. Twelve subjects (7 males; 5 females; age 25±4), with informed consent, were recruited for the study. The heart function assessments were carried out using a 3.0T MR scanner (Siemens, Germany). The heart rate was monitored using ECG. Ten short axis slices covering the whole heart from apex to base were imaged using a free-breathing 2D real-time SSFP, to which the Karhunen-Loeve transform filter was applied along the temporal direction to increase the signal-to-noise ratio. The imaging parameters were as follows: slice thickness 8 mm with a 2 mm gap, field of view (FOV) = 340 × 287 mm^2^, pixel spacing = 2.25 mm/pixel, repetition time/echo time (TR/TE) = 2.5/1.1 ms, matrix size = 160 × 128, TPAT=4, bandwidth=1488 Hz/pixel, temporal resolution = 59.5 ms, and cine duration of 5 s for each slice, containing 84 frames covering end-expiration and end-inspiration. The ED and ES frames in end-expiratory stage of each slice of twelve subjects were used to validate the final network model. The segmentation contours for the LV, LV myocardium, and RV of each frame were provided by the radiologist. The ED and ES frames in other respiratory stages of each slice were used for testing.

### 2.2. 2017 ACDC Data

The data was obtained from the automated cardiac diagnosis challenge (ACDC) data [20], which was initiated at the 2017 MICCAI Segmentation Challenge in the STACOM workshop. It consists of 150 subjects with normal, previous myocardial infarction, dilated cardiomyopathy, hypertrophic cardiomyopathy, and abnormal right ventricle. The data is divided into the training and testing sets, with 100 and 50 cases, respectively. As the training set contains the LV, LV myocardium, and RV contours, we used the training set to determine the parameters for segmentation in our study.

### 2.3. Data Processing

The ACDC data varied in size from 154 × 224 to 428 × 512. We resized all images to 160 × 128 by bilinear interpolation without any image cropping operations. Since the data acquisition from different imaging acquisition sequences can introduce inconsistencies in image intensity and pixel intensity, the 16-bit images were normalized to 8-bit images. Thereafter, contrast-limited adaptive histogram equalization (CLAHE) [38] was used to enhance the contrast of the grayscale image. Finally, 1902 images (100 subjects) from ACDC data were used for training, and 80 images (4 subjects) and 160 images (8 subjects) from free-breathing CMR data were used for validation and testing, respectively. For the ground truth (labeled image) in the training stage, LV blood pool, LV myocardium, RV blood pool, and background are labeled as 4, 3, 2, and 1, respectively.

## 3. Methods

### 3.1. Outline of the Method

**Figure 2:**
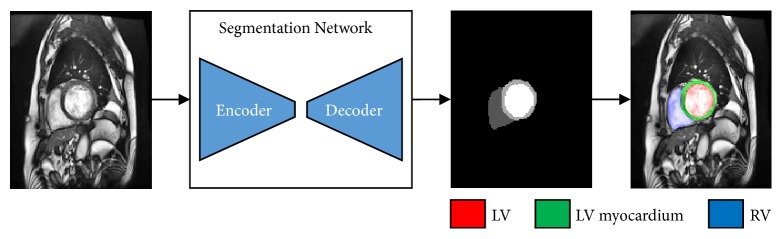
The block diagram of the proposed method for segmentation.

The block diagram of the proposed method is shown in Figure 2. In our method, the proposed segmentation network can be divided into two stages: encoder and decoder. The encoder stage was used for CMR image representation and pixel-level classification, and a decoder stage was used to restore the original spatial resolution. To better display the segmentation result, red, green, and blue colors indicate the region of LV, LV myocardium, and RV in Figure 2.

#### 3.1.1. Heart Segmentation

**Figure 3:**
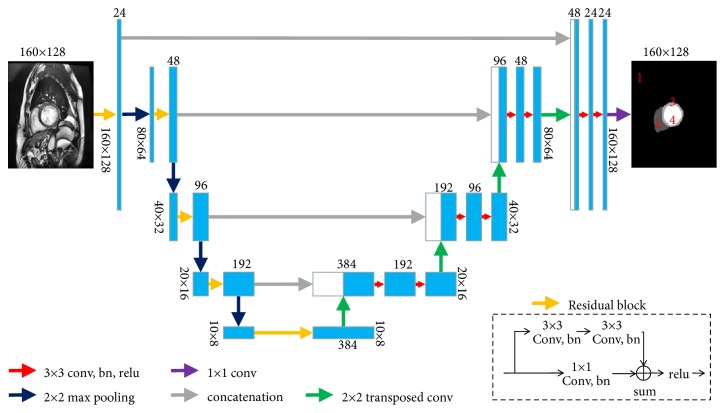
The proposed network architecture for heart segmentation. The label value of LV, LV myocardium, RV, and background is 4, 3, 2, and 1, respectively.

For LV, LV myocardium, and RV segmentation, it was always necessary to initially locate the heart region when using older methods [7–9, 30], which is highly time-consuming especially for the deep learning methods. We proposed a deep learning network based on U-Net and ResNet [39] to directly locate and segment the heart region.  Figure 3 demonstrates the architecture of the network for heart segmentation. The architecture consists of a down-sample path (encoder) followed by an up-sample path (decoder) to restore the size of the input image. In the down-sample path (left section in Figure 3), the input image is 160 × 128 in size. The residual block includes two 3 × 3 convolution layers and one 1 × 1 convolution layer which are appended by batch normalization (BN) and subsequently by ReLU activation (see Figure 3). The max pooling of size 2 × 2 is used to down-sample the convolved maps. The dropout layer is used in the bottom of the convolution layer to prevent overfitting. The dropout ratio was set to 0.5. In the up-sample path (right section in Figure 3), the 2 × 2 transposed convolution was used to up-sample the convolved maps. Several skip connections were used to concatenate feature maps between down-sample and up-sample paths, which could provide more feature information for localization and segmentation. A 1 × 1 convolution layer maps the 24 feature channels to 4 classes. The pixel value in the output image of 4 to 1 indicates the LV, LV myocardium, RV, and background, respectively. Thereafter, the loss function layer is used to calculate the loss value from the output of the SoftMax layer.

#### 3.1.2. Loss Function

Previous methods used Dice loss [40] to solve the problem of class imbalance between heart structure and background in cardiac image segmentation. Recently, Sudre et al. presented an improved Dice loss, called generalized Dice loss [41, 42], which is a robust and accurate loss function for unbalanced tasks and is formulated as

**Figure 4:**
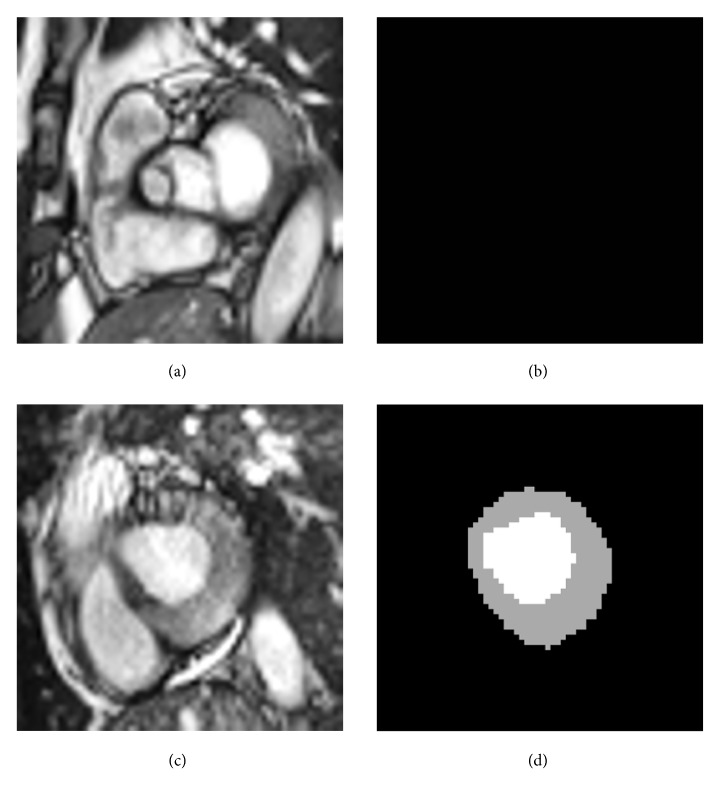
Original images in ACDC data (a, c) and corresponding labeled images (b, d).

(1) Loss=1−2∑k=1Kwk∑m=1MYkmTkm∑k=1Kwk∑m=1MYkm2+Tkm2

(2) wk=1∑m=1MTkm2

where *Y* and *T* are predicted label image and ground truth, respectively, *K* is the number of classes, *M* is number elements along the first two dimensions of *Y* or *T*, and *w*_*k*_ is a weighting factor for each class. In our study, some images in the ACDC data did not have full labels, as illustrated in Figure 4. In such cases, *T*_*km*_=0 and *w*_*k*_=infinite. To avoid this kind of problem, the loss and *w*_*k*_ are revised and given by

(3) Loss=1−2∑k=1Kwk∑m=1MYkmTkm+ε∑k=1Kwk∑m=1MYkm2+Tkm2+ε

(4) wk=1∑m=1MTkm,Tkm≠0

where *ε*=10^−8^ is used to avoid the numerical issue of dividing by 0. When *T*_*km*_=0, the weight of *k* class is determined by the *Y*_*km*_; it could improve the performance of segmentation.

### 3.2. Implementation Details

**Figure 5:**
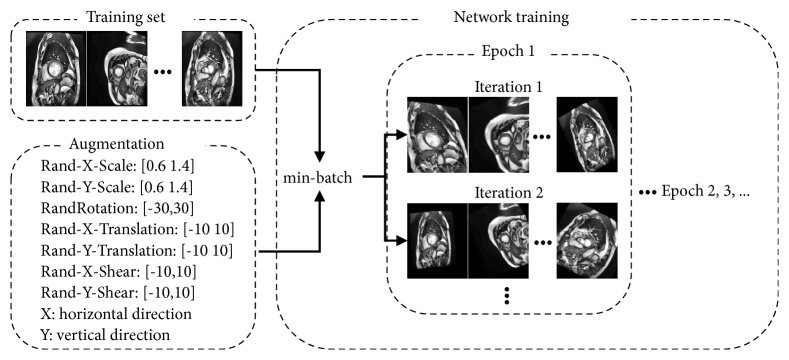
The process of data augmentation in training.

The heart segmentation network was implemented in Matlab 2019a using a deep learning toolbox and trained on a computer with Nvidia RTX 2080Ti (11GB memory). In the training stage, 1902 images from ACDC data were used for training, and 80 images from free-breathing CMR data were used for validation. In order to prevent the network from overfitting, affine geometric transformation (scaling, rotation, shearing, and translation), gaussian noise, gaussian blur, and elastic deformations were used to augment the training set before training in cardiac images segmentation [24, 30, 43]. However, random augmentation before training increased the training time; therefore we used a random augmentation data strategy during the training process. For each iteration of training, a random combination of transformations was applied on the minibatch images and was not stored in memory. Therefore, different images were used for network training in each iteration as it could help prevent overfitting, especially for data that come from different acquisition protocols.  Figure 5 shows the process of data augmentation in training for heart segmentation.

The network was trained by decreasing the proposed loss function using the adaptive moment estimation (ADAM) optimizer [44]. The initial learning rate of 10^−3^ was decayed by 0.98 per epoch, where the minibatch included 16 images. The network weights were initialized using He initialization [45]. L2 regularization weight decay of 10^−4^ was added to the loss function to reduce overfitting. Besides, for using random augmentation of the minibatch, the training set was also shuffled in each epoch before training. Since each minibatch was different due to the random augmentation, the learning rate was restored to 10^−3^ every 100 epochs. We used the Dice curve of the minibatch to observe the training process for training and validation sets. The training of the model was discontinued when no improvement in the Dice score was seen (at about 200 epochs). For the present network, it takes about 6 hours to complete the training of 200 epochs.

### 3.3. Evaluation Criterion

To evaluate the performance of the developed methods, the Dice and intersection over union (IoU) were calculated and compared with the ground truth. The Dice for each class is given by

(5) $$\begin{array}{c} Dice=2\frac{Y_{k}\cap T_{k}}{Y_{k}+T_{k}} \end{array}$$

where *Y*_*k*_ and *T*_*k*_ are predicted and ground truth image of each class (*k*=2, 3, 4). The IoU for each class is defined by

(6) $$\begin{array}{c} IoU=\frac{Y_{k}\cap T_{k}}{Y_{k}\cup T_{k}} \end{array}$$

## 4. Experiments and Results

### 4.1. Comparison of the Learning Curves of the Proposed Method and U-Net

**Figure 6:**
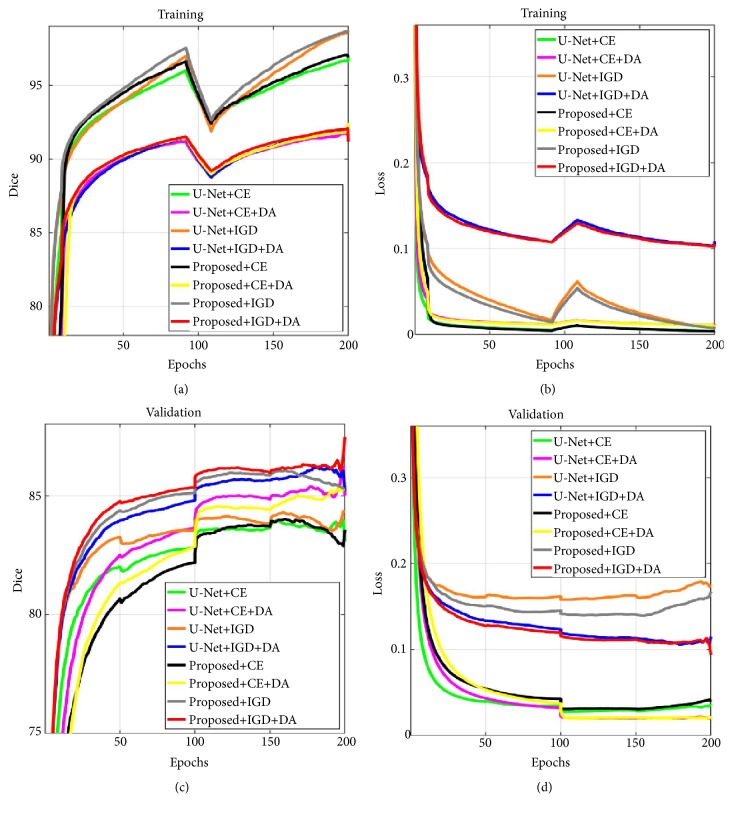
Comparison of the learning curves of proposed network and U-Net with cross-entropy (CE) loss, improved generalized Dice (IGD) loss, and data augmentation (DA). Dice curve (a, c) and loss curve (b, d) of training and validation sets, respectively. Note that the learning curves have been smoothed by moving average method.

We trained the proposed network and U-Net by using the same training scheme: the learning rate was restored to 10^−3^ every 100 epochs using the ADAM solver. Other network training settings were the same as described in Section 3.2, and for every epoch the model was evaluated on the validation set.  Figure 6 shows the Dice and loss curve of two networks using cross-entropy (CE) loss, improved generalized Dice (IGD) loss, and data augmentation (DA). To observe the learning curves, the validation loss of the proposed model and U-Net with DA decreased consistently with a decrease in training loss, indicating less overfitting on the training set. The convergence speed of both models with IGD loss was faster than CE loss for the validation curve. Furthermore, the proposed network using IGD loss and DA shows the fastest convergence and the lowest loss value when compared to the U-Net model.

### 4.2. The Result of Segmentation on Validation and Testing Set

**Figure 7:**
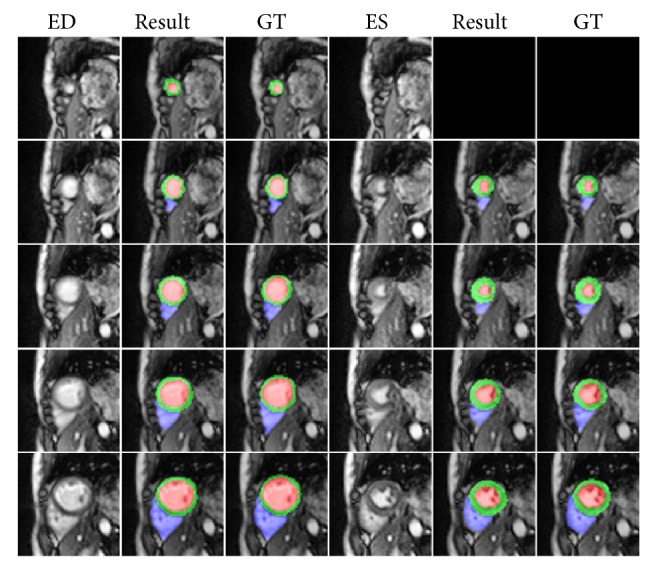
Representative ED and ES frames segmentation results by proposed method from slice 1 to slice 5 in short axis. Red, green, and blue indicate LV, LV myocardium, and RV, respectively. Note that the results have been cropped for better observation of the heart region (GT: ground truth).

**Figure 8:**
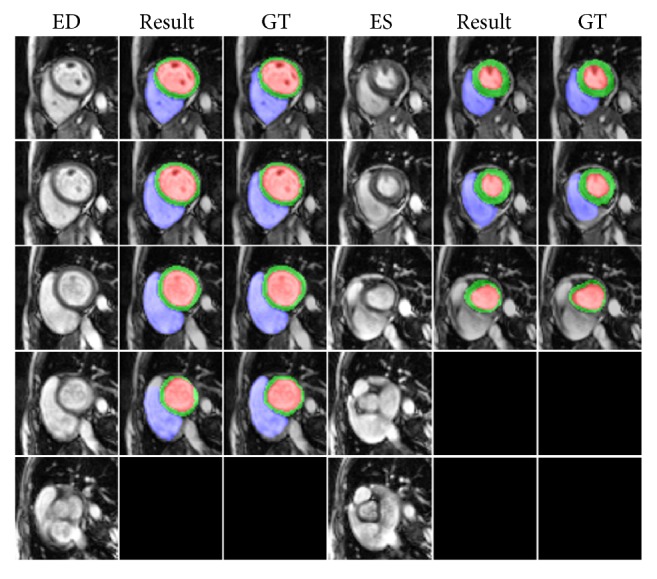
Representative ED and ES frames segmentation results by the proposed method from slice 6 to slice 10 in short axis. Red, green, and blue indicate LV, LV myocardium, and RV, respectively (GT: ground truth).

**Figure 9:**
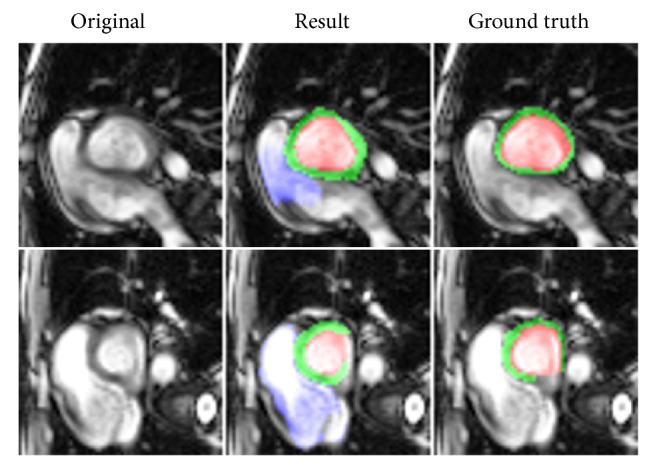
Failure cases from the heart segmentation model.

The highest mean Dice score model was selected for segmentation and evaluation. Figures 7 and 8 show the representative segmentation results by the proposed method from apex to basal slices of ED and ES frames on the testing set. Our method gave accurate results for most slices; however, some failure cases were found in basal slices since these slices included other structures such as the pulmonary artery, left ventricle, and right ventricle outflow tract, among other vessels, as demonstrated in Figure 9. The segmentation performances of the proposed network and U-Net on validation and testing sets are summarized and compared in Table 1. The proposed model and U-Net using IGD loss had a better Dice and IoU score than CE loss. Compared to those without DA, both models with DA showed a significant improvement in validation and testing sets. Moreover, the proposed network with IGD and DA showed the best performance in the evaluation of Dice and IoU score.

### 4.3. The Performance in Different Heart Cycles

**Figure 10:**
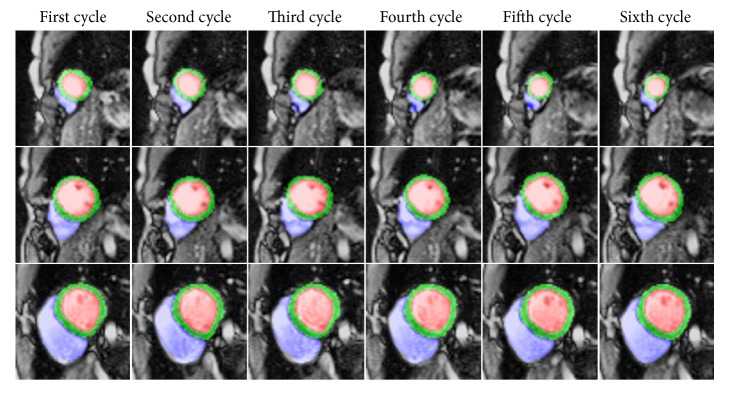
Segmentation results of six cycles at ED frames in different slices. The first row is near the apex slice, the second row is mid-slice, and the third row is near basal slice.

Accurate segmentation at ED or ES frames in different heart cycles could help radiologists obtain information about respiratory variations in cardiac motion [2]. Since the testing dataset in other cycles did not provide labeled contours of ED and ES frames, we only used our model to segment LV, LV myocardium, and RV and observed the performance of segmentation.  Figure 10 shows the segmentation results of the ED frames in different heart cycles. Our approach obtained better segmentation results of LV and LV myocardium, which are the same as the ED frames of the end-expiratory stage. The segmentation results in different cycles of RV were slightly different due to the influence of respiratory motion.

## 5. Discussion

Previously, some studies pointed out that the use of data from different imaging protocols could better assess the performance of deep learning algorithms [20]. Inspired by this problem, we presented a novel deep learning network based on U-Net and ResNet for heart segmentation using data from two imaging protocols in our study. The proposed method used a random augmentation data strategy for training. In each iteration of training, a random combination of transformations was applied to the minibatch and was not stored in the program, a strategy that could reduce training time. Moreover, we proposed an improved Dice loss to improve the accuracy of segmentation. The proposed method was fully automated for heart segmentation without requiring any prior knowledge, and the network produced segmentation results at roughly 10 images per second.

Since the deep learning approaches based on U-Net were widely used in CMR image segmentation [24, 26, 29, 37], we selected it as the baseline method to compare with our method. The proposed network required a lower number of learnable parameters (4.4 million) as compared to U-Net (30 million, when initial number of filters was 64 and BN was used) in the study. The designed residual block helped to alleviate the problem of vanishing gradients and improve the performance of feature extraction. Due to the limited free-breathing CMR data, we used ACDC data as a training set and a random augmentation data strategy during the training to solve the problem of lack of data. These images underwent random combination of transformation in each iteration. In this study, the training images approached 377 000 after 200 epochs when the minibatch consisted of 16 images. When using the data augmentation before training strategy, it was very time-consuming to train a large amount of data. However, a large amount of training data helps to train a better model and reduce overfitting. Compared to the data augmentation before training strategy, the training time was much shorter when training the same epochs. The proposed loss function reduced the impartation of class imbalance. In comparison to cross-entropy loss functions, the improved loss function obtained higher Dice and IoU scores. Furthermore, our network has a lower model complexity, which is easy to implement and train own model.

For the reason of no definitive approach to design hyperparameters, such as the number of layers, filter size, and learning rate in the design of the deep learning framework, in our study we selected hyperparameters based on exhaustive methods and then turned to experiments. For example, we tried several initial learning rates ranging from 10^−2^ to 10^−3^, and after observing the learning curve, an initial learning rate of 10^−3^ was selected due to the best Dice curve of the validation set. However, the Dice curve did not go up after 100 epochs. We tried to adjust the learning rate to the initial learning rate every 100 epochs using the ADAM optimizer. The validation Dice increased consistently as the training progressed. These training schemes helped the model to reach the highest accuracy of segmentation on the validation set. Although several studies had proved the residual network with a better performance in feature extraction [39], we found that too many residual blocks lead to poor segmentation results in our research. Therefore, we only used one residual block before each max pooling layer. The training time of the improved network was the same as standard U-Net, and the segmentation accuracy was higher according to the experiment results.

A limitation of this work was the basal slice segmentation. This slice included the pulmonary artery, left ventricle and right ventricle outflow tract, and other structures. Compared to other slices, the basal slice occupied a small part of ACDC data. Even with data augmentation, it was hard to improve accuracy using the proposed method. Even for experts, segmenting the basal slice is challenging [20]. Other studies have also reported the failure of the basal slice with deep learning methods [20, 28, 46, 47]. Furthermore, the anatomical structure of ED and ES frames was different due to the influence of respiratory motion in different cardiac cycles and could produce poo segmentation results, especially for supervised deep learning methods. Existing solutions to these problems are to exclude the basal slice in CMR imaging or the use of a larger database, e.g., the UK Biobank [48], might help to enhance segmentation accuracy. Further studies should include the simultaneous cardiac function quantification and segmentation, and so on [49, 50].

## 6. Conclusion

In this paper, we proposed a fully automatic heart segmentation approach based on deep learning. The designed residual block and improved loss function were used to improve the segmentation performance of the LV, LV myocardium, and RV. A random data augmentation strategy was applied to reduce the training time and alleviate the problem of overfitting. The results showed that the presented method has high segmentation accuracy and stability. It is worth pointing out that it is the first report employing a one-stage deep learning method for the segmentation of free-breathing CMR data. In the future, we aim to develop more methods and test on a larger sample of free-breathing imaging data.

## Figures and Tables

**Table 1:** Comparing the segmentation performance of the proposed network and U-Net on validation and testing set (IoU: intersection over union, CE: cross-entropy, IGD: improved generalized Dice, DA: data augmentation, and italic formatting indicating the best Dice and IoU score for each class).

Loss function	LV Dice		LV Myocardium Dice		RV Dice	
	valid	test	valid	test	valid	test
U-Net +CE	0.763	0.815	0.632	0.690	0.675	0.680
U-Net +CE+DA	0.867	0.880	0.757	0.782	0.758	0.746
U-Net +IGD	0.784	0.835	0.676	0.722	0.741	0.684
U-Net +IGD+DA	0.870	0.876	0.752	0.771	0.768	0.788
Proposed+CE	0.765	0.815	0.646	0.701	0.722	0.704
Proposed+CE+DA	0.823	0.875	0.704	0.770	0.767	0.748
Proposed+IGD	0.804	0.817	0.668	0.711	0.730	0.670
Proposed+IGD+DA	*0.878*	*0.919*	*0.768*	*0.806*	*0.795*	*0.818*

	LV IoU		LV Myocardium IoU		RV IoU	
	valid	test	valid	test	valid	test

U-Net +CE	0.697	0.758	0.517	0.585	0.606	0.615
U-Net +CE+DA	0.798	0.827	0.640	0.679	0.702	0.694
U-Net +IGD	0.712	0.772	0.558	0.615	0.677	0.620
U-Net +IGD+DA	0.791	0.816	0.618	0.661	0.715	0.734
Proposed+CE	0.709	0.760	0.531	0.592	0.651	0.639
Proposed+CE+DA	0.753	0.822	0.590	0.669	0.710	0.697
Proposed+IGD	0.733	0.757	0.550	0.602	0.659	0.605
Proposed+IGD+DA	*0.802*	*0.860*	*0.642*	*0.699*	*0.741*	*0.761*

## Data Availability

The datasets used and analyzed during the current study are available from the corresponding author upon reasonable request.
